# Germ-line mutations in epidermal growth factor receptor (*EGFR) *are rare but may contribute to oncogenesis: A novel germ-line mutation in *EGFR *detected in a patient with lung adenocarcinoma

**DOI:** 10.1186/1471-2407-11-172

**Published:** 2011-05-16

**Authors:** Irene Centeno, Pilar Blay, Iñigo Santamaría, Aurora Astudillo, Ana S Pitiot, Fernando G Osorio, Patricia González-Arriaga, Fernando Iglesias, Primitiva Menéndez, Adonina Tardón, Jose M Freije, Milagros Balbín

**Affiliations:** 1 Laboratorio de Oncología Molecular, Instituto Universitario de Oncología del Principado de Asturias, Hospital Universitario Central de Asturias, (Celestino Villamil s/n), Oviedo, (33006), Spain; 2 Unidad de Cáncer Familiar, Servicio de Oncología Médica, Instituto Universitario de Oncología del Principado de Asturias, Hospital Universitario Central de Asturias, (Celestino Villamil s/n), Oviedo, (33006), Spain; 3 Servicio de Anatomía Patológica, Hospital Universitario Central de Asturias, (Celestino Villamil s/n), Oviedo, (33006), Spain; 4 Banco de Tumores, Instituto Universitario de Oncología del Principado de Asturias, Hospital Universitario Central de Asturias. (Celestino Villamil s/n), Oviedo, (33006), Spain; 5 Departamento de Bioquímica y Biología Molecular, Instituto Universitario de Oncología del Principado de Asturias, Universidad de Oviedo, (Campus del Cristo), Oviedo, (33006), Spain; 6 Unidad de Epidemiología Molecular, Instituto Universitario de Oncología del Principado de Asturias, Facultad de Medicina, Universidad de Oviedo, (Campus del Cristo), Oviedo, (33006), Spain

## Abstract

**Background:**

A subset of lung cancer patients harbour *EGFR *somatic mutations in their tumours and are candidates for treatment with EGFR tyrosine kinase inhibitors. In a few cases *EGFR *mutations have also been found in the germ line, suggesting a role in lung carcinogenesis. Objetives of this study were: 1) To analyze the *EGFR *gene mutations in a population diagnosed with lung adenocarcinoma from Northern Spain. 2) To determine the frequency of a new germ-line mutation found in our laboratory as well as the frequency in our population of three other EGFR germ-line mutations detected by other authors. 3) To determine whether the novel mutation detected may have a functional effect on the EGFR protein.

**Methods:**

Tumour DNA samples were obtained from frozen or paraffin embedded tumour tissues. Samples of DNA from peripheral blood cells were obtained from 912 individuals with lung cancer recruited from the CAPUA study [1,2], 477 unrelated healthy donor individuals and 32 individuals with other types of cancer. *EGFR *gene exons 18 to 21 were studied by direct standard dideoxy sequencing. Specific mutations were determined either by direct sequencing or by specific RFLP analysis. Cell lines were transfected with *EGFR*-mutant plasmids and analysed by western blot with antibodies specific for total or phosphorylated-EGFR.

**Results:**

We found EGFR mutation in 12 of the 71 tumour samples (17%). One tumour contained two mutations. One mutation (p.R776G) was present as a germ line. Using an RFLP analysis, this mutation was not found in 954 alleles from healthy individuals studied, concluding that it is not a polymorphism. The mutation was not found either in genomic DNA from 912 lung cancer patients. Three additional EGFR germ-line mutations that were already described were not found in any of the studied samples. These observations show that *EGFR *mutated alleles are rare in the population. *In vitro *studies revealed that tyrosine autophosphorylation is enhanced in p.R776G-mutant EGFR when compared with wild-type EGFR. This enhanced autophosphorylation in the absence of ligand may be associated with a proliferative advantage.

**Conclusions:**

Germ-line mutations in EGFR are rare but may contribute to oncogenesis

## Background

One of the main goals of cancer research is to find a way of selecting the appropriate patients for specific therapies. A promising result regarding lung cancer was the discovery that somatic activating mutations in the tyrosine kinase domain of the *EGFR *gene identified a subset of non-small cell lung cancer (NSCLC) patients that may respond to tyrosine kinase inhibitors [3-5]. After this seminal discovery, evidence accumulated in recent years has supported the idea that patients harbouring these mutations in their tumours show response to EGFR tyrosine kinase inhibitors [6,7]. Based on the results of the latest phase III trials [8,9] gefitinib, an EGFR tyrosine kinase inhibitor, has been approved in Europe for first line treatment of adult patients with locally advanced or metastatic NSCLC carrying activating *EGFR *somatic mutations. Thus, introduction of mutational screening of somatic mutations into clinical care is already a requirement for taking therapeutic decisions [10].

As recorded in the Sanger Institute Catalogue of Somatic Mutations in Cancer data-base (COSMIC v49 Release) [11,12], curated from the recent literature, a total of 22489 samples from lung cancer tumours have been analyzed for *EGFR *tyrosine kinase domain mutations. The number of samples with mutations is 4722, with 5055 mutations detected, being p.L858R missense mutation and in frame-deletions in exon19 the most frequently detected alterations in *EGFR*. Resequencing studies provide an opportunity to find novel variants that may be involved in the activity of the protein, and may also allow us to find germ line alterations that support implication of these genes in familial carcinogenesis.

In the course of our *EGFR *mutational screening in lung adenocarcinoma patients from the area of Asturias, Northern Spain, we found a tumour sample with a double somatic mutation in *EGFR *gene; one of the changes was demonstrated to be present as a germ-line mutation. We have determined the frequency in our population of the new germ-line mutation as well as the frequency of three additional *EGFR *germ-line mutations described earlier, and we have investigated the impact of the novel mutation on the biochemical properties of the EGFR protein.

## Methods

### Tissue procurement

Lung adenocarcinoma samples were obtained from frozen or paraffin embedded tumour tissues and their paired normal tissues deposited in the Tumour Bank of Hospital Universitario Central de Asturias (HUCA). Samples were evaluated for tumour content prior to use for nucleic acid extraction. RNA and DNA were purified using Trizol method (Invitrogen) following manufacturer's instructions. In addition, genomic DNA was obtained from peripheral blood cells of 912 individuals with lung cancer recruited from the CAPUA study [1,2], 477 unrelated healthy donor individuals aged 21-65 years recruited through the Blood Bank of HUCA and 32 individuals with other types of cancer. All samples were obtained with informed consent. The study was approved by the ethical committee of the hospital and the research was done conformed to the Helsinki Declaration.

### Mutational analyses

*EGFR *exons 18 to 21 were amplified from genomic DNA and *EGFR *tyrosine kinase domain was amplified from cDNA when possible. Standard sequencing PCR was set up with kit *Big Dye Terminator*^® ^v1.1 (Applied Biosystems) and analyzed in an *ABIPrism310 *(Applied Biosystems). Primers used for the different PCRs are listed in Table 1.

**Table 1:** Primers used for EGFR PCR analysis

Oligonucleotide	Sequence 5' to 3'
**1 **(EGFR Exon 18R)	TCCCCACCAGACCATGAGAG
**2 **(EGFR Exon 19R)	GAGGTTCAGAGCCATGGACC
**3 **(EGFR Exon 20F)	CCATGAGTACGTATTTTGAAACTC
**4 **(EGFR Exon 20R)	CATATCCCCATGGCAAACTCTTGC
**5 **(EGFR Exon 18F)	CCTGCTGGGCCATGTCTGGC
**6 **(EGFR Exon 19F2)	GTGCATCGCTGGTAACATCC
**7 **(EGFR Exon cDNA)	CGAAGGCGCCACATCGTTC
**8 **(EGFR Exon cDNA)	GAGGAGATCTCGCTGGCAG
**9 **(EGFR Exon 21F2)	CCTGGCATGAACATGACCC
**10 **(EGFR Exon 21R2)	AATACAGCTAGTGGGAAGGC
**11 **(EGFR Exon 20F)	GTGGAGGTGAGGCAGATGCC
**12 **(EGFR Exon 20F)	GCGAAGCCACACTGACGTG
**13 **(EGFR Exon 19F)	CCTGAGGTTCAGAGCCATGG
**14 **(EGFR Exon 19R FAM*)	*GGACTCTGGATCCCAGAAGG
**15 **(EGFR Exon cDNA)	GAGGACCGTCGCTTGGTGCA
**16 **(EGFR Exon 21R Hex*)	*CAATACAGCTAGTGGGAAG
**17 **(EGFR Exon 21 ASO L858R)	CAAGATCACAGATTTTGGACG

### Detection of specific mutations in EGFR exons 20 and 21

*EGFR *exon 20 p.R776G mutation and exon 21 p.V843I and p.P848L were detected by specific RFLP analysis since nucleotide changes affect restriction sites for *Hae*III, *Rsa*I and *Pst*I respectively. All restriction fragments were visualized on a 2% agarose gel. If no matching restriction enzyme could be found, samples were analyzed by direct sequencing. In addition, high sensibility mutation detection methods were also developed to perform a mutational screening study for the most frequent *EGFR *activating mutations. Briefly, an *EGFR *exon 19 hot spot region was amplified from 50 ng of genomic DNA using primers 13 and 14 (Table 1). A 24 cycle-PCR was done in standard conditions with 10 μmol of fluorescent reverse primer and 20 μmol of the direct one, in a final volume of 50 μL. Products were analyzed through capillary electrophoresis in an automatic sequencer *ABIPrism310 *(Applied Biosystems) and software *GenMapper*. For detection of the p.L858R mutation, an allele specific oligonucleotide multiple PCR was performed. Primers 15 and 16 amplify the region encompassing the mutation; the reverse primer is marked with 5'HEX. Primer 17 is 858R allele-specific. A multiple 26-cycle PCR was done in standard conditions using 10 μmol of direct and reverse primers and 40 μmol of the allele-specific primer, in a final volume of 50 μL. Products were analyzed through capillary electrophoresis the same way as exon 19 deletions.

### Construction of expression vectors encoding mutant EGFR

Vectors for mammalian expression containing human *EGFR *were kindly donated by W. Pao (Memorial Sloan-Kettering Cancer Center, NY). Plasmids containing p.R776G mutation were constructed by inserting a *Bst*XI fragment obtained by PCR amplification of an EGFR segment from a cDNA carrying this mutation into a *Bst*XI-digested pcDNA3-EGFR vector. All constructs were fully sequenced to confirm the absence of unwanted additional mutations in the *EGFR *region.

### Cell transfections and western blot analysis

HEK293T and COS-7 cells were transiently transfected, using Lipofectamine™ LTX with PLUS™ (Invitrogen), with the indicated plasmids and with a vector containing the green fluorescent protein to check transfection efficiency by fluorescence microscopy. Forty-eight hours after transfection, lysates of these cells were used to quantify levels of phosphorylated EGFR. Total cell protein, extracted using RIPA buffer, were resolved by SDS-PAGE (8%) in parallel duplicated gels, transferred to nitrocellulose membranes and incubated with primary antibody ((Phospho-EGFR Tyr-1045, or total EGFR; Cell Signalling Technology) diluted 1/1000 in 5% BSA in TBS-T overnight at 4°C. Loading control gels were run in additional electrophoresis gels, blotted to nitrocellulose membrane and incubated with antibodies to beta-actin. Membranes incubated with primary antibody were washed and incubated 1 hour at RT with peroxidase labelled goat antirabbit antibody 1/2000 diluted in TBS-T containing 5% non-fat milk. Signals were visualized with ECL system (Amersham Biosciences) and lumino-image analyzer LAS3000 (Fujifilm), following the manufacturer's instructions.

## Results

### Analysis of EGFR gene mutations in a lung cancer population from Northern Spain

We selected a group of 62 frozen lung adenocarcinomas and their corresponding normal matching tissue from patients that were subjected to surgery from years 2001 to 2006 at HUCA and completely sequenced *EGFR *exons 18 to 21. In addition, 9 samples from transbronchial biopsies were also analyzed for mutational status of the *EGFR *Tyrosine kinase domain. Results of the detected mutations are shown in Table 2. The obtained results showed mutation in 12 of the 71 tumour samples (17%), one of which contained two mutations. Regarding smoking status, gender and mutation presence, 7 out of 13 detected mutations were found in lung tumours from women; 6 of them were non smokers and the remaining one was a former smoker. In the male group, 6 mutations were detected, 4 of which corresponded to non smokers and the 2 remaining mutations were detected in the same patient, a smoker male. Therefore, in our cohort, *EGFR *mutations are more frequent in women and patients who have never smoked, as reported in previous studies [13].

**Table 2:** Distribution of EGFR somatic mutation's frequency from lung adenocarcinoma tissues and transbronchial biopsies, depending on gender and smoking condition

		EGFR mutations		
**Sex**	**Smoking status**	**Exon 19**	**Exon 20**	**Exon 21**

Male (n = 55)	Yes (n = 47)		**1 **(p.R776G)	**1 **(p.L858R)
	
	No (n = 8)	**2 **(p.E746_A750del) **1 **(p.E746_S752del)		**1 **(p.L858R)

Female (n = 16)	Yes (n = 7)	**1 **(p.E746_A750del)		
	
	No (n = 9)	**2 **(p.E746_T751del) **1 **(p.E746_S752delinsV)	**1 **(p.D761_E762insEAFQ)	**2 **(p.L858R)

### Detection of a novel germ-line mutation and population frequency study

One of the tumour samples harboured two coexistent *EGFR *mutations c.2326C > G p.R776G in exon 20 and c.2573T > G p.L858R in exon 21. Analysis of matched non tumoral tissue and available DNA from peripheral blood revealed that one of the mutations (p.R776G) was present as a germ-line alteration, since it was present both in the tumoral and non tumoral tissue as well as in genomic DNA obtained from peripheral blood. This sample belonged to a 47 year-old man with a 50 pack-year smoking history, who was diagnosed with a stage IIIA (pT3N2M0) undifferenciated lung adenocarcinoma. He was initially treated with surgery, but died of metastatic disease one year later. Genealogical information of this patient revealed that one of his siblings died of cancer but unfortunately no samples were available to study and no other healthy family members were requested to be analyzed, for ethical reasons.

To determine if this germ-line mutation was a polymorphism present in the population, we analysed 477 available healthy donor individuals using a RFLP analysis with *Hae*III restriction enzyme. The mutation was not found in 954 alleles from control individuals studied. We also searched for the presence of this mutation in genomic DNA from another group of 912 lung cancer patients, 285 of them with familial cancer history and a cohort of 32 patients with other types of cancer. We did not find any additional sample harbouring the p.R776G *EGFR *mutation as a germ-line alteration, so it can be concluded that p.R776G mutation is a rare event.

Three more *EGFR *germ-line mutations have been recently described: p.T790M [14,15] in exon 20, and p.V843I and p.P848L in exon 21 [16,17]. We analyzed the frequency of these germ-line mutations in a set of 285 samples of our group from NSCLC patients with familial cancer antecedents. Table 3 shows the samples analyzed for each mutation. None of these mutations were detected in our studied population, neither the control population nor the group of samples from lung cancer patients. It can be concluded that p.R776G, as well as p.T790M, p.V843I and p.P848L are rare mutant *EGFR *alleles.

**Table 3:** Number of patients analyzed for p.R776G, p.T790M, p.V843I and p.P848L mutation presence in genomic DNA

	Analyzed samples						
**Mutation**	**NSCLC + FCH + SS**	**NSCLC + FCH + NSS**	**NSCLC + NFCH + SS**	**NSCLC + NFCH + NSS**	**Other**	**Healthy donors**	**Total**

**p.R776G**	268	17	596	31	32	477	**1421**
**p.T790M**	268	17					**285**
**p.V843I**	268	17		31			**316**
**p.P858L**	268	17		31			**316**

NSCLC: non small cell lung cancer; FCH: familiar cancer history; NFCH: no familiar cancer history; SS: smoking status; NSS: non smoking status; Other: patients with other types of cancer.

### In vitro EGFR p.R776G analysis

**Figure 1:**
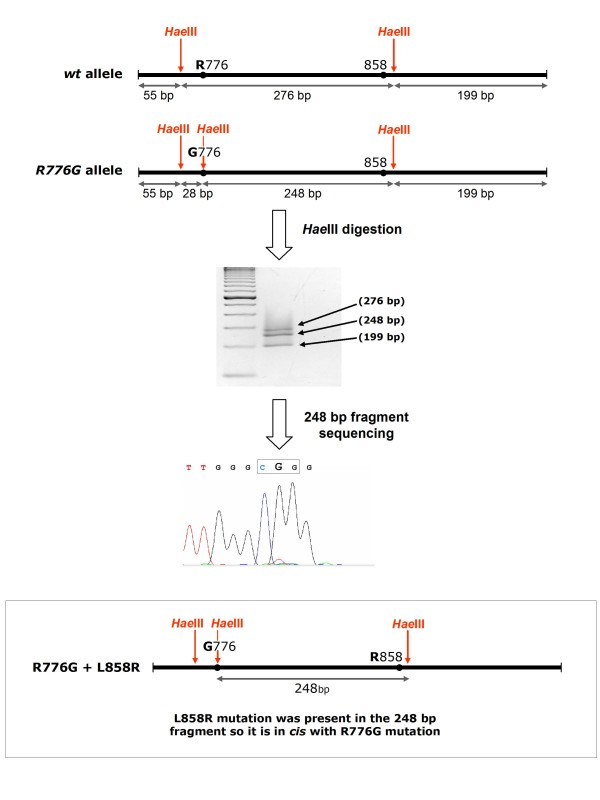
**Strategy developed to resolve if p.R776G and p.L858R were located *in cis***. Taking into account that c.2326C > G mutation causing p.R776G amino acid change generates a new *Hae*III restriction site, when an exon 21 PCR fragment containing these mutations was digested with *Hae*III, two different bands could be distinguished from the heterozygous tumour sample: one 276 bp fragment (wt for the position c.2326) and one 248 bp fragment (mutated at this position). Sequence analysis of 248 bp fragment revealed that the p.L858R mutation was present in the same band as the p.R776G mutation.

To determine if both *EGFR *alleles were affected in the tumour sample where a double mutation was found, we first determined if both mutations were located in *cis *or in *trans*. Figure 1 depict the strategy developed to determine if p.R776G and p.L858R were located in the same strand of DNA. Thus, taking into account that c.2326C > G mutation causing p.R776G amino acid change generates a new *Hae*III restriction site, when an exon 21 PCR fragment containing these mutations was digested with *Hae*III, two different bands could be distinguished from the heterozygous tumour sample: one 276 bp fragment (wt for p.R776) and one 248 bp fragment (mutated for p.R776G). Sequence analysis of both fragments revealed that the base change yielding an Arg at position 858 was present in the same band as the mutation yielding Gly at position 776. It was concluded then that both mutations were located in *cis*, that is, they occurred in the same allele.

**Figure 2:**
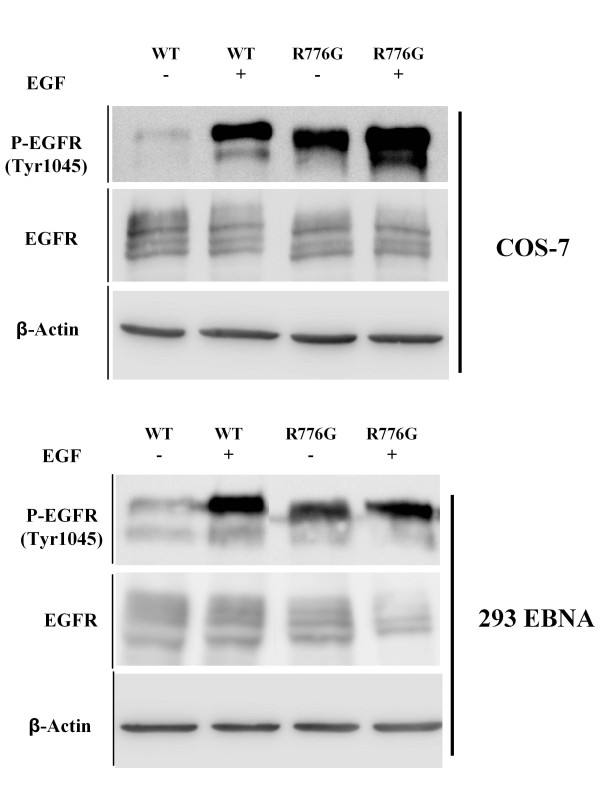
**Anti-Phospho-EGFR immunoblot analysis**. COS-7 and 293 EBNA cells were transiently transfected as described. All EGFR constructs are expressed at similar levels. EGFR activity (phospho-EGFR level) is increased in p.R776G mutant, both in presence or in absence of ligand.

EGFR mutant vector constructions were performed based on a pcDNA3.1 plasmid containing human wild-type EGFR cDNA; the EGFR tyrosine kinase domain was replaced with the corresponding p.R776G mutant variant and the resultant construction was used to transfect 293EBNA and COS7 cells. Forty-eight hours after transfection cell lysates were analyzed by western blot using primary antibodies anti Phospho-EGFR. In vitro analyses showed that in the absence of ligand (EGF), an enhanced tyrosine autophosphorylation can be observed in cells transfected with the plasmid containing EGFR-R776G when compared to those transfected with wild-type EGFR (Figure 2).

## Discussion

In recent years, mutational screening and re-sequencing of potential oncogenes in tumours allowed the identification of hot spots for mutations and also new genes not previously involved in tumour genesis [12,18]. Identification of somatic mutations in exons coding for tyrosine kinase domain of *EGFR *in tumours from patients with NSCLC and their association with response with targeted therapies, has led to the introduction of mutational screening of tumours in the clinic. A substantial number of tumours have already been studied and in addition to somatic mutations, few germ line mutations have been found.

In this work we have described the identification of a new germ line mutation p.R776G in *EGFR *in a patient suffering from NSCLC. In order to rule out that this mutation could be a polymorphism, we analysed 954 alleles from healthy donors and did not find this variation. In addition, we have searched for the presence of this mutation in 285 genomic DNA samples from patients with lung cancer and some history of familial cancer, and 627 samples from patients with lung cancer and no familial cancer antecedents. We have not found this mutation in any other patient. Three other germ line mutations in *EGFR *have been described: the first is p.T790M, that has been found in at least five families [14,15,19,20], p.V843I, described in two cases [17,21] and p.P848L [22]. It is interesting to mention that p.T790M was the first mutation in NSCLC associated with resistance to therapy with tyrosine kinase inhibitors [23-25]. We also studied if these mutations were present in our cohort of individuals with lung cancer and familial history of cancer but we did not find any of them. Moreover, the sequencing of the entire exon 20 of *EGFR *did not reveal any other alteration in this gene. Vikis *et al *[26] also analyzed a number of families with high susceptibility to lung cancer for the presence of p.T790M mutation and did not find this alteration in any of 237 probands. Girard *et al *[20] found p.T790M as germ-line in 2 of 369 cases of never smokers with NSCLC, both patients with a family history significant for lung cancer. Concerning p.V843I and p.P848L no studies with a large group of samples was performed previously to the present work. From these results, we can conclude that p.R776G, p.T790M, p.V843I and p.P848L are rare *EGFR *alleles that have been found in germ line from patients suffering lung cancer.

Regarding the possibility that these mutations can represent cancer susceptibility alleles, several studies have been performed on p.T790M allele (reviewed by Suda *et al *[27]). Its detection as a somatic mutation in tumours supports this option. Moreover, this mutation has also been found together with the activating p.L858R mutation, both in tumours and in cell lines that never were in contact with tyrosine kinase inhibitor treatments, suggesting that p.T790M might be also growth promoting. Bell *et al *[14] observed that this mutation seems to occur in *cis *with the p.L858R activating mutation. Kinase activity of EGFR p.T790M mutant has been reported as indistinguishable from wild-type EGFR [23,25], but Vikis *et al *[26] reported that p.T790M mutation alone causes increased phosphorylation levels. Godin-Heymann *et al *have shown that, although p.T790M mutation has a moderate effect on EGFR function, when combined with p.L858R or p.746_750del show a remarkable enhancement of EGFR activity [28]. Finally, animal models generated to inducibly express the p.T790M mutation have been also shown to develop lung tumours [29]. The latency of developing tumours is longer in those mice with p.T790M mutation alone than those bearing both p.T790M and p.L858R.

In relation to p.R776G, the novel germ line mutation revealed in this work, some lines of evidence similar to the p.T790M mutation would also support its possible role as a cancer susceptibility allele. Mutations at this position in the *EGFR *gene have also been discovered in tumours as somatic mutations [30]. In the tumour sample analyzed in this work, the p.R776G mutation was found together with a p.L858R activating somatic mutation in *cis*. A double mutation p.R776G/p.L858R was found by Wu *et al *[30] and the patient harbouring these two mutations did not respond to tyrosine-kinase inhibitor therapy. Alterations at R776 position giving rise to a different amino acid substitution (p.R776C) have also been described as somatic mutations and in conjunction with p.L858R [5,31]. Preliminary studies by transfection of cell lines with *EGFR *mutant constructs revealed that in the absence of ligand (EGF), an enhanced tyrosine autophosphorylation can be observed in cells harbouring the plasmid containing EGFR-R776G when compared with wild-type EGFR, providing biochemical evidence of the functional relevance of this mutation.

Further analysis encompassing single and double mutant constructs will help to clarify the role of these alterations on EGFR function and in tumour genesis. It is tempting to speculate that, since p.R776G has appeared as germ-line mutation, it would have a weaker effect on EGFR functions than the double mutations detected within the tumours. Functional analyses of these mutants need to be evaluated by cellular assays, for example with tagged constructs [32], by transformation assays and by tumorigenicity studies in animal models. It will also be interesting to test the sensibility of these mutants to TK inhibitors in order to gain information of clinical value.

Mutations in genes coding for proteins with tyrosine kinase activity have been one of the genetic alterations most frequently detected in cancer over the last years [33]. Pathways activated by these proteins usually lead to proliferative advantages, thus they have received much consideration, given their association with malignant proliferation. Regarding the role of receptor tyrosine kinase alterations in familial carcinogenesis, germ line mutations in *KIT *have been described in familial gastrointestinal stromal tumours (GIST), *RET *mutations are involved familial medullary thyroid cancers and other endocrine cancer predisposition syndromes and *MET *mutations have been found in papillary renal cancer syndrome among others [34]. Somatic mutations in these genes have also been described in sporadic tumours and in the case of GIST, activating mutations in *KIT *are predictors of response to targeted therapy with imatinib [35-37].

Taking these examples as precedent, it is reasonable to hypothesize that germ-line mutations in the *EGFR *gene could be involved in lung cancer pathogenesis, and probably in other tumours in whose development EGFR deregulation can play a role. Although there have been reported some evidences of mendelian inheritance in the pathogenesis of lung cancer [38], the intrinsic genetic factors controlling susceptibility to this malignancy might be hidden by strong environmental factors like cigarette smoking and air pollution, implicated in the mutagenesis of many other genes controlling pathways involved in the development of these tumours [18,39].

More studies are needed in large cohorts of cancer patients with familiar history of cancer in order to link *EGFR *germ-line mutations to development of the disease, but these results and precedent observations do suggest that *EGFR *is a candidate to be involved in familial oncogenesis.

## Conclusions

Germ-line mutations in *EGFR *gene are rare but may contribute to oncogenesis.

## Competing interests

The authors declare that they have no competing interests.

## Authors' contributions

IC: carried out mutational analysis, genetic studies and plasmid constructions. PB: participated in design of the study, analysis of clinical data and helped in draft the manuscript. AA, PM: performed pathological review and selections of all tumour samples and analyses of clinical data. IS, ASP, FI: participated in mutational analysis. FGO, JMF: carried out transfections and western blot analyses and participated in review of the manuscript. PGA, AT: provided samples and clinical data. MB: designed the study and drafted the manuscript. All authors approved the final manuscript.

## Pre-publication history

The pre-publication history for this paper can be accessed here:

http://www.biomedcentral.com/1471-2407/11/172/prepub
